# Intraspecific variation and phenotypic plasticity of olive varieties in response to contrasting environmental conditions

**DOI:** 10.1093/aobpla/plaf060

**Published:** 2025-11-13

**Authors:** Siham Wakib, Ahmed El Bakkali, Hayat Zaher, Abdelilah Meddich, Cherkaoui El Modafar, Frédéric Lagane, Sylvain Delzon, Karim Barkaoui, Eric Garnier

**Affiliations:** Laboratoire d'Excellence d'Agrobiotechnologie et Bioingénierie, Centre AgroBiotech, Unité de Recherche Labellisée CNRST (URL05-CNRST), Université Cadi Ayyad, Av Abdelkrim El Khattabi, BP 511, 40000 Marrakech, Morocco; CEFE, Univ Montpellier, CNRS, EPHE, IRD, Montpellier, France; INRA, Centre Régional de la Recherche Agronomique de Meknès, UR Amélioration des Plantes et Conservation des Ressources Phytogénétiques, BP 578, Route Haj Kaddour, VN 50000 Meknès, Morocco; INRA, Centre Régional de la Recherche Agronomique de Marrakech, Unité de l’Amélioration des plantes et de la Qualité, BP 533, 40000 Marrakech, Morocco; INRA, Centre Régional de la Recherche Agronomique de Meknès, UR Amélioration des Plantes et Conservation des Ressources Phytogénétiques, BP 578, Route Haj Kaddour, VN 50000 Meknès, Morocco; INRA, Centre Régional de la Recherche Agronomique de Marrakech, Unité de l’Amélioration des plantes et de la Qualité, BP 533, 40000 Marrakech, Morocco; Laboratoire d'Excellence d'Agrobiotechnologie et Bioingénierie, Centre AgroBiotech, Unité de Recherche Labellisée CNRST (URL05-CNRST), Université Cadi Ayyad, Av Abdelkrim El Khattabi, BP 511, 40000 Marrakech, Morocco; Laboratoire d'Excellence d'Agrobiotechnologie et Bioingénierie, Centre AgroBiotech, Unité de Recherche Labellisée CNRST (URL05-CNRST), Université Cadi Ayyad, Av Abdelkrim El Khattabi, BP 511, 40000 Marrakech, Morocco; University of Bordeaux, UMR BIOGECO, INRAE, Pessac, France; University of Bordeaux, UMR BIOGECO, INRAE, Pessac, France; CIRAD UMR AMAP, Montpellier, France; AMAP, Univ Montpellier, CIRAD, CNRS, INRAE, IRD, Montpellier, France; CEFE, Univ Montpellier, CNRS, EPHE, IRD, Montpellier, France; Section (and Section Editor): Form & Function

**Keywords:** intraspecific variation, *Olea europaea* L, phenotypic plasticity, phenotypic traits, plant resource use, trait–environment relationships

## Abstract

Assessing the extent of genotypic and phenotypic trait variation within a genetically diversified species is crucial to understanding how plants cope with environmental differences. We examine these components in *Olea europaea* L. *europaea*, one of the most widespread and diverse tree crops cultivated in the Mediterranean Basin, a region facing rapid climatic shifts with increasing summer drought. We compared trait values of 83 olive varieties from different Mediterranean countries, grown in two *ex situ* varietal collections with contrasting environments: subhumid and semi-arid climates. Ten leaf-, stem- and branch traits related to resource and water use were compared across 50 varieties within each site, and phenotypic plasticity was assessed for the 17 varieties common to them. Trait plasticity was assessed with the phenotypic dissimilarity index while varietal plasticity was assessed in multidimensional trait space with the multivariate plasticity index. Our results showed considerable phenotypic variability within (up to 59.54%) and between (up to 39.17%) sites. Varieties grown in semi-arid conditions were more conservative, showing denser leaves and wood, and thicker bark. Common varieties exhibited contrasting degrees of plasticity across traits, demonstrating that high plasticity for some traits does not necessarily imply overall plasticity. Additionally, varieties with conservative trait values were not less plastic than more acquisitive varieties. Varieties showed distinct phenotypic adjustments across sites, with trait variations indicating acclimation strategies to reduce water loss in the arid environment. Our results also suggest that acclimation to different environments occurs through the adjustment of multiple traits, complicating plasticity comparisons across varieties.

## Teaser

This study explores how cultivated olive trees adjust their leaf, stem, and branch traits across two contrasting environments. By examining 17 varieties in subhumid and semi-arid sites, we reveal substantial phenotypic variation and trait-specific plasticity. Some traits are highly plastic while others remain stable, showing that plant acclimation involves coordinated adjustments across multiple traits, highlighting the interplay of genetic background and environment in shaping adaptive strategies.

## Introduction

Intraspecific trait variation, which plays a key role in modulating trait-environment relationships (Lepš et al. 2011, Violle et al. 2012, Siefert et al. 2015, Kitagawa et al. 2022), involves genetic variation, phenotypic plasticity and ontogenetic changes. Differences in trait values among individuals of the same species can result from genetic variation and adaptations to their geographical or environmental conditions of origin (Hereford 2009). In addition, phenotypic plasticity, defined as the ability of a single genotype to produce different phenotypes across environments through differential gene expression (Linhart and Grant 1996, Valladares et al. 2007), is another major source of trait variation. Fox et al. (2019), Stotz et al. (2021) and Cavallaro et al. (2023) emphasise the importance of plant phenotypic plasticity as a key acclimation strategy in response to rapid climatic variations. However, uncertainties remain regarding the factors controlling both inter- and intra-specific phenotypic variability (Stotz et al. 2022).

Changes in trait values in response to pedo-climatic variations have been studied both across species (e.g. Niinemets 2001, Moles et al. 2014) and within species (e.g. Weemstra et al. 2021, Welles and Funk 2021), but the patterns are not always consistent for several reasons. As discussed by e.g. Vellend et al. (2014) and Anderegg (2023), genetic and physiological constraints can limit intraspecific variation, and the ecological significance of a single trait can vary considerably from one species or variety to another. These factors lead to complex trait-environment relationships, calling for specific analyses of the acclimation strategy at each level of organisation. Anderegg (2023) suggests that a better understanding of trait-environment relationships at the intraspecific level requires (i) controlled environment/common gardens studies to disentangle phenotypic plasticity and genotypic variation, and (ii) the exploration of trait responses across different organs and organisational levels of the organism.

Here, we directly address a significant and persistent research gap in the trait literature by investigating these patterns in cultivated varieties of olive (*Olea europaea* L. subsp. *europaea* var. *europaea*), a widely cultivated and economically important tree species, recognised as a drought resistant species (Fernandez 2014 and references therein), related to its sclerophyllous nature and its typically conservative resource use compared to many other crops (Garnier et al. 2021). The olive tree is one of the most widespread cultivated species in the Mediterranean Basin, which notably represents the primary centre of its cultivation and diversification (Besnard et al. 2018). It is characterized by a large genetic diversity, with at least 1200 cultivated varieties described (Bartolini et al. 2005), associated with a large phenotypic variation (Ganino et al. 2006), mostly described by morphological characteristics of the different organs (especially leaves and fruits), which are primarily used for varietal identification. This reservoir of diversity can be considered as an asset in the context of the current climate change affecting the Mediterranean Basin. This region is currently undergoing an accelerated transition to more arid zones (MedECC 2020), characterised by increased drought stress resulting from a modified balance between the evaporative demand of the atmosphere and the availability of water in the soil. The remarkable genotypic and phenotypic diversity of olive, coupled with its wide geographical distribution across the Mediterranean Basin, presents a unique opportunity to explore the role of different components of intraspecific variability in plant response to the environment.

Due to their relevance for plant carbon and water economies, the responses of leaf and stem traits to climatic conditions have been particularly studied in trees (e.g. Rosas et al. 2019, Fajardo et al. 2024, Kassout et al. 2024). Leaf size and morphology play a central role in leaf energy balance, with large leaves often being disadvantageous in hotter, drier, and high-irradiance environments (Wright et al. 2017 and references therein). In arid sites, plants tend to have tough leaves, with high leaf mass *per* area (LMA) and leaf dry matter content (LDMC) (Niinemets 2001, Wright et al. 2004, Poorter et al. 2009), while trends for leaf thickness are less clear and vary from one study to another (Poorter et al. 2009, Garnier et al. 2019). For stems, a high wood density has been found to be associated with increased resistance to drought-induced xylem cavitation (Chave et al. 2009, Fajardo et al. 2022). In addition, a large sapwood-to-leaf area ratio is indicative of a low transpiring area per unit area of stem water-conducting tissue (Tyree and Zimmermann 2002). This, combined with low specific stem length, induced by a high wood density, is expected under drier conditions, as it should improve water use at the canopy level. Finally, it has been suggested that bark thickness, through its role in water storage and embolism repair, should be higher in drier environments (Rosell 2019); however, this remains to be formally tested. Some of these traits are structured along so-called functional dimensions, including the leaf- and the wood economics spectrum (LES and WES, respectively). The LES captures a trade-off between rapid resource acquisition and efficient resource conservation. Fast-return leaves combine short lifespans, high photosynthetic and respiration rates, and low structural investment, whereas slow-return leaves have the opposite characteristics. The WES describes a similar continuum and captures trade-offs between fast-growing, low-density wood and slow-growing, mechanically resistant wood (Reich et al. 1992, Wright et al. 2004, Chave et al. 2009, Díaz et al. 2016, Stotz et al. 2022). Understanding the extent and the patterns of this phenotypic plasticity and trait variation under diverse environmental conditions is therefore crucial. It would not only shed light on ecological acclimation, but would also provide valuable insights for plant breeding in the context of future drier climates (Brooker et al. 2022). In olive (*O. europaea* L.), breeding is particularly challenging due to its long juvenile phase, high heterozygosity, and predominant vegetative propagation, which limit genetic recombination (Valverde et al. 2024). Despite these constraints, breeding programmes have focused on traits such as early bearing, fruit size, oil content, and disease resistance (León et al. 2015, Valverde et al. 2023). Recent studies have also highlighted the importance of incorporating wild olive germplasm to enhance resilience to biotic and abiotic stresses (Díaz-Rueda et al. 2020). Therefore, assessing intraspecific variation in phenotypic traits is crucial for identifying the traits that can be targeted in breeding programmes to improve adaptation to changing climates.

In this study, we aimed to assess the intraspecific variation in 83 olive varieties originating from different Mediterranean countries and maintained in two varietal collections with very contrasting pedo-climatic conditions, one in France and the other in Morocco. We evaluated genetic differentiation by comparing trait values between varieties within each collection and assessed phenotypic plasticity for a subset of 17 common varieties between the two collections. We expand on recent studies assessing the role of various phenotypic traits for drought adaptation responses in olive (e.g. Trentacoste et al. 2018, Kassout et al. 2024, Terral et al. 2025), asking the following questions: (i) What is the extent of phenotypic variability in olive in response to contrasting pedo-climatic conditions, and how much of this variability is explained by genetic differences among varieties and phenotypic plasticity? We expect that genetic variation between varieties will contribute more than plasticity to explain trait variability, (ii) Does phenotypic plasticity differ among varieties and among traits? We hypothesise that plasticity will vary significantly not only among varieties, but also for the different traits measured. Furthermore, we expect that morphological traits, such as leaf size, which are often less structurally and metabolically constrained, will exhibit greater plasticity than structural traits (Nicotra and Davidson 2010), (iii) Does plasticity in individual traits (univariate plasticity) reflect overall plasticity (multivariate plasticity)? Here we hypothesise that high plasticity in a single trait does not necessarily translate into high plasticity across multiple traits. In this framework, we define a ‘plastic variety’ as one that shows relatively greater trait adjustment across sites compared to other varieties. Finally, (iv) do olive varieties with relatively conservative trait values, as assessed by traits of the LES and WES, exhibit lower plasticity than varieties with relatively acquisitive trait values (Stotz et al. 2021, Cavallaro et al. 2023)? Indeed, it has been suggested that ‘slow’ plants are less plastic than ‘fast’ plants due to their higher tissue structural investment (Stotz et al. 2021, Carrascosa et al. 2023, but see, e.g. Garnier et al. 2019). We extend the hypothesis to the WES, and expect that varieties with a high wood density, presumably with a low stem and leaf hydraulic conductance and a low leaf photosynthetic capacity (Reich 2014), will be less plastic than those with a low wood density.

## Materials and methods

### Study sites

The study was conducted on trees established in two *ex situ* olive collections (Table 1). The first one is the French Olive Germplasm Bank (‘subhumid site’ hereafter), located on the island of Porquerolles, in the south of France, managed by the Conservatoire Botanique National Méditerranéen (CBNMed). At this site, the bioclimate is classified as meso-Mediterranean, characterized by subhumid conditions according to Daget (1977), with a mean annual precipitation of 605 mm and a De Martonne aridity index of 22.5 (Table 1). The subhumid site includes 193 varieties (Khadari and Pinatel 2018) with trees planted in rows 5 × 7 m apart (1.7 ha, 2 plots). Each variety is represented by two to three trees, which are grouped within the same plot and distributed randomly within rows. The second collection, the World Olive Germplasm Bank of Marrakech (‘semi-arid site’ hereafter), is located at the Tassaout experimental station (INRA Marrakech, Morocco). As summarized in Table 1, this site experiences considerably lower precipitation (238 mm) and a higher mean annual temperature (19°C), resulting in a distinctly arid climate according to the Köppen–Geiger classification (Kottek et al. 2006). A drip irrigation system is used to compensate for the low rainfall (Table 1). Based on the De Martonne aridity index, calculated using precipitation and irrigation as supplementary water supply, this site falls within the semi-arid range. The collection includes 329 olive varieties from 14 different Mediterranean countries identified based on a previous study by El Bakkali et al. (2019), with trees planted in rows 7 × 4 m apart (7 ha, 3 plots). Each variety is represented by two to three trees located within the same plot: in the first plot, trees of the same variety were planted side by side, whereas in the second and third plots they were distributed randomly. Additionally, the sites differ in their soil characteristics, as detailed in Supplementary Table S1.

**Table 1.** Geographic coordinates, irrigation regime, and climatic characteristics (1994–2023) of the two studied olive collections, including mean annual temperature (MAT) and mean annual precipitation (MAP).

	Latitude, longitude	Altitude (m)	Irrigation (mm)	MAT (°C)	MAP (mm)	I (mm)	Climate classification
Subhumid site^a^	42°59′30″N, 6°12′14″E	18	None	16.9	605.2	22.5	Subhumid
Semi-arid site^b^	31°49′10″N, 07°25′58″W	614	224	19.1	238	15.88	Semi-arid

Climate classification was based on the De Martonne aridity index (I) calculated as MAP_(+ water supply)_/(MAT + 10) (de Martonne 1926). This index allow categorization into arid (<10), semiarid (10-20), subhumid (20-30), and humid (>30) climate types.

^a^Data obtained from the meteorological station located 2 km northeast of the site; ^b^Data obtained from the Ouled Nacer station located 16 km away from the site (31°45′17.0″N, 7°22′58.6″W).

### Selection of trees

**Figure 1.**
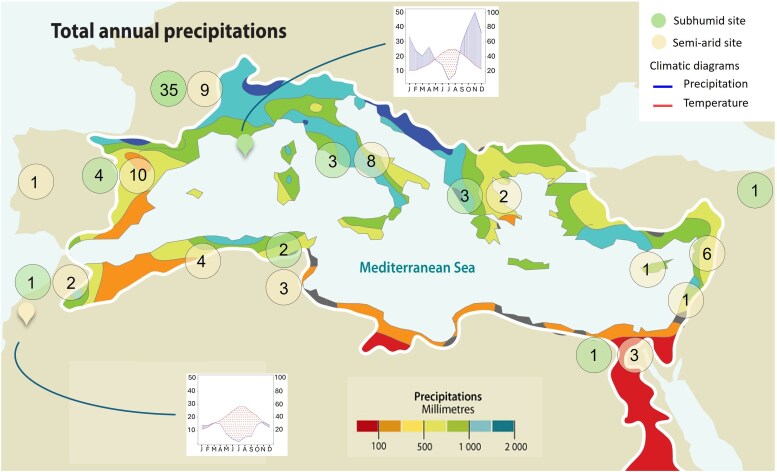
Map of the Mediterranean region illustrating the spatial distribution of mean annual precipitation across the Mediterranean region and the number of olive varieties studied by country of origin, with location markers indicating the two study sites. Climatic diagrams corresponding to each site are included, with mean monthly temperature (left axis) and precipitation (right axis), and a dotted area indicating the drought period [monthly precipitation <2 times average monthly temperature (Gaussen and Bagnouls 1952)]. Base map adapted from ‘Total annual precipitations’ by GRID-Arendal, used under CC BY-NC-SA 2.0 (https://creativecommons.org/licenses/by–nc). Source: https://www.grida.no/resources/5909.

Fifty varieties were selected and screened for phenotypic traits in each collection, among which 17 were common to both collections (referred to as ‘common varieties’ hereafter), while 33 were distinct to each of them (referred to as ‘distinct varieties’ hereafter). To maximize phenotypic diversity, varieties were selected from a large range of geographic origins. The semi-arid site comprised a set of varieties originating from 12 Mediterranean countries, while the set of varieties in the subhumid site, although primarily French (37 varieties), also included 13 varieties from 6 other Mediterranean countries and Iran (Fig. 1). This selection also ensured representation of the different genetic backgrounds previously identified by Khadari and El Bakkali (2018) and El Bakkali et al. (2019) (see Supplementary Tables S2 and S3).

In the subhumid site, trees were planted between 1979 and 1991, while in the semi-arid site, planting dates ranged from 2003 to 2009. Trunk diameter was measured at 50 cm above ground level. The average trunk diameter was 21.79 ± 6.81 cm and 19.09 ± 4.24 cm (mean ± standard deviation) for the subhumid and semi-arid sites, respectively. The similar trunk diameter across sites suggests that trees have reached a similar size. All trees were adult, fruit bearing individuals (above 15–20 years old at the minimum), indicating that they have all reached a comparable development stage.

### Trait measurements

Measurements were conducted between 2019 and 2022 in the subhumid site and in 2023 in the semi-arid site, on mature leaves and current-year growth stems. Two to three trees were studied for each variety, with samples collected from the upper, outer, and well-lit parts of the tree canopy, specifically from the southwest and southeast sides.

Ten phenotypic traits were chosen for their relevance to the plant’s carbon and water economies (Table 2; cf. Pérez-Harguindeguy et al. 2013).

**Table 2.** Description of phenotypic traits studied: units of measurement and functional significance.

Trait	Unit	Functional significance	References
Leaf area (LA)	cm^2^	Energy and water balance	Cornelissen et al. (2003) and Wright et al. (2017)
Leaf width (LW)	cm	Photosynthesis optimization	Givnish (1987)
Leaf Length to Leaf Width ratio (LL:LW)	cm cm^−1^	Thermoregulation and mechanical support	Nicotra et al. (2011)
Leaf mass per area (LMA)	g m^−2^	Leaf economics spectrum	Wright et al. (2004)
Leaf Dry Mass Content (LDMC)	mg g^−1^	Investment in support and defence structures	Wilson et al. (1999)
Leaf thickness (LT)	µm	Structural protection of the photosynthetic tissues	Pérez-Harguindeguy et al. (2013)
Specific stem length (SSL)	cm g^−1^	Light interception	Poorter et al. (2012)
Sapwood area to twig leaf area ratio (*A*_S_:*A*_L_)	cm^2 ^m^−2^	Water balance	Tyree and Zimmermann (2002) and Mencuccini et al. (2019)
Branch wood Density (BWD)	g cm^−3^	Biomechanical support and xylem resistance to embolism	Hacke et al. (2001) and Chave et al. (2009)
Relative bark thickness (RBT)	mm mm^−1^	Resistance to fire and water storage	Rosell and Olson (2014) and Rosell et al. (2023)

### Leaf traits

Ten to twelve one-year-old leaves per variety were collected. Immediately after being cut from the branch, the leaves were placed into a test tube filled with deionized water to submerge the petioles. They were then put in a cool box before being transferred to a refrigerator for at least 12 h to ensure full hydration (Garnier et al. 2001). After removing the petiole, leaf fresh mass (LFM) and leaf thickness (LT) of the fully hydrated leaves were measured respectively using a precision balance and a digital micrometre (Digimatic IP65 (0–25 mm), Mitutoyo, Kawasaki, Japan) at four different points on the leaf lamina. Leaf length (LL) and width (LW) were also recorded to assess the leaf form index, as the ratio of leaf length to leaf width (LL:LW). Leaves were then scanned to determine the leaf area (LA) using the *ImageJ* software (version 1.54d). Finally, the leaf dry mass (LDM) was determined after the leaves were oven-dried for at least 48 h at 60°C. The LMA was calculated as the ratio of LDM to LA, and the leaf dry matter content (LDMC) as the ratio of LDM to LFM.

### Stem and branch traits

Six twigs, each with two annual segments (*n* and *n* − 1 years), were sampled for each variety at the same location as for the leaf sampling. The measurements were conducted on the most recent part of the stem. After removing the bark, two perpendicular diameter measurements of each twig were taken using a digital calliper, from which the twig area ‘cross-sectional sapwood area’ (*A*_S_) was calculated. This area is considered a reliable proxy for the vessel cross-sectional area for small twigs, which is typically used to calculate the ‘Huber value’ (Huber 1928). All leaves on each twig were scanned to obtain the twig leaf area (*A*_L_) using the *imageJ* software (version 1.54d), and the sapwood area to twig leaf area ratio (*A*_S_:*A*_L_) was then calculated (Mencuccini and Bonosi 2001). The length and dry mass of each twig were finally measured to calculate the specific stem length (SSL) defined as the ratio of stem length to stem dry mass.

Branch wood density (BWD) was determined on six branches *per* variety. Six cm long segments with a diameter between 0.8 and 1.5 cm were sampled from each branch. Wood density analyses were conducted using X-rays (Polge 1966). Before X-ray imaging, the samples were conditioned in a climatic chamber with 12% humidity for one week to ensure uniform residual humidity of the samples. For each sample, a 2 mm-thick slice was cut. X-ray radiographs of the wood sections were obtained using an ISOVOLT 3003 industrial radiography generator equipped with a V4N X-ray tube (GE Inspection Technologies GmbH, Ahrensburg, Germany). The radial density profiles obtained in duplicate *per* section were recorded on Ready Pack II M100 films (Carestream INDUSTREX, Rochester, USA). Finally, the average BWD was calculated using Windendro software (Guay et al. 1992, Torres-Ruiz et al. 2019). The final density values were determined as the mean density-weighted by thickness, previously measured using a digital micrometre (Digimatic MDQ-30 (0–30 mm), Mitutoyo, Kawasaki, Japan). Images of branch cross sections were also used to determine the relative bark thickness (RBT) using *ImageJ* software (version 1.54d). Four measurements of bark thickness and two measurements of branch diameter, taken from two perpendicular sides, were recorded. The RBT was then calculated as the ratio of the average bark thickness (multiplied by 2) to the average branch diameter (Rosell et al. 2014, Midgley and Lawes 2016).

### Statistical procedures

All statistical analyses were performed using the R software version 4.4.0 (R Core Team 2024; https://www.r-project.org/).

Three different one-way ANOVAs were performed using variety-level means to test the differences in leaf, stem and branch trait values: (i) between the two sites across all 83 varieties to evaluate the overall trait variability, (ii) between the 17 common varieties and the 33 distinct varieties at each site to assess whether trait variation was comparable for the two groups of varieties, and (iii) between the two sites across only the 17 common varieties to evaluate the overall trait plasticity. A two-way ANOVA was then performed using tree-level means to investigate more specifically the ‘site × variety’ interaction for the common varieties.

To quantify the relative contributions of genetic variation and phenotypic plasticity to overall trait variability, we calculated the percentage of variance explained by each component using the sum of squares from one-way ANOVA results. Variance components include genetic variation, corresponding to the overall differences among varieties across both sites, with variety as a factor; phenotypic plasticity, corresponding to the differences between the common varieties, with site as a factor; and residual variance, representing variation within varieties (i.e. among trees).

To assess the phenotypic plasticity of the 17 common varieties between the two sites, we calculated the phenotypic dissimilarity index (PhD) for each trait using the R function described by Puglielli et al. (2022). The PhD quantifies the intraspecific variability of standardized traits between trees growing under contrasting environmental conditions. It has been calculated as the average pairwise phenotypic dissimilarity between all individual trees of the same variety between sites while considering trait variability among trees within each site. The PhD index ranges from 0 (no plasticity) to 1 (maximum plasticity) (Puglielli et al. 2022). The average plasticity for each trait was calculated using the PhD values of all varieties combined, and the difference between traits was tested using an ANOVA followed by a Tukey’s Honest Significant Difference (Tukey HSD) post-hoc test to assess significance.

The multivariate plasticity index (MVPi) was calculated for the common varieties as the average Euclidean distances of the trait values of all individual trees of the same variety between sites, based on their projections in the first three principal components of PCA (Pennacchi et al. 2021). This index considers the covariations among traits. PCAs were conducted within each site, to explore the phenotypic space defined by the ten traits and classify the varieties along the fast-slow axis reflecting their resource use strategy, from acquisition to conservation. A varimax rotation was performed (using the varimax() function from the ‘psych’ package; Revelle 2017) to maximize factor loadings on the identified axis. Then, using the projections of individual trees, the average score of each variety on the fast-slow axis was calculated for each site. These scores were then compared with the PhD index of the different traits and the MVPi index using the Pearson correlation coefficients. This allowed us to assess whether varieties with ‘fast’ or ‘slow’ strategies were significantly associated with variations in plasticity.

## RESULTS

### Trait variation within and between sites

**Figure 2.**
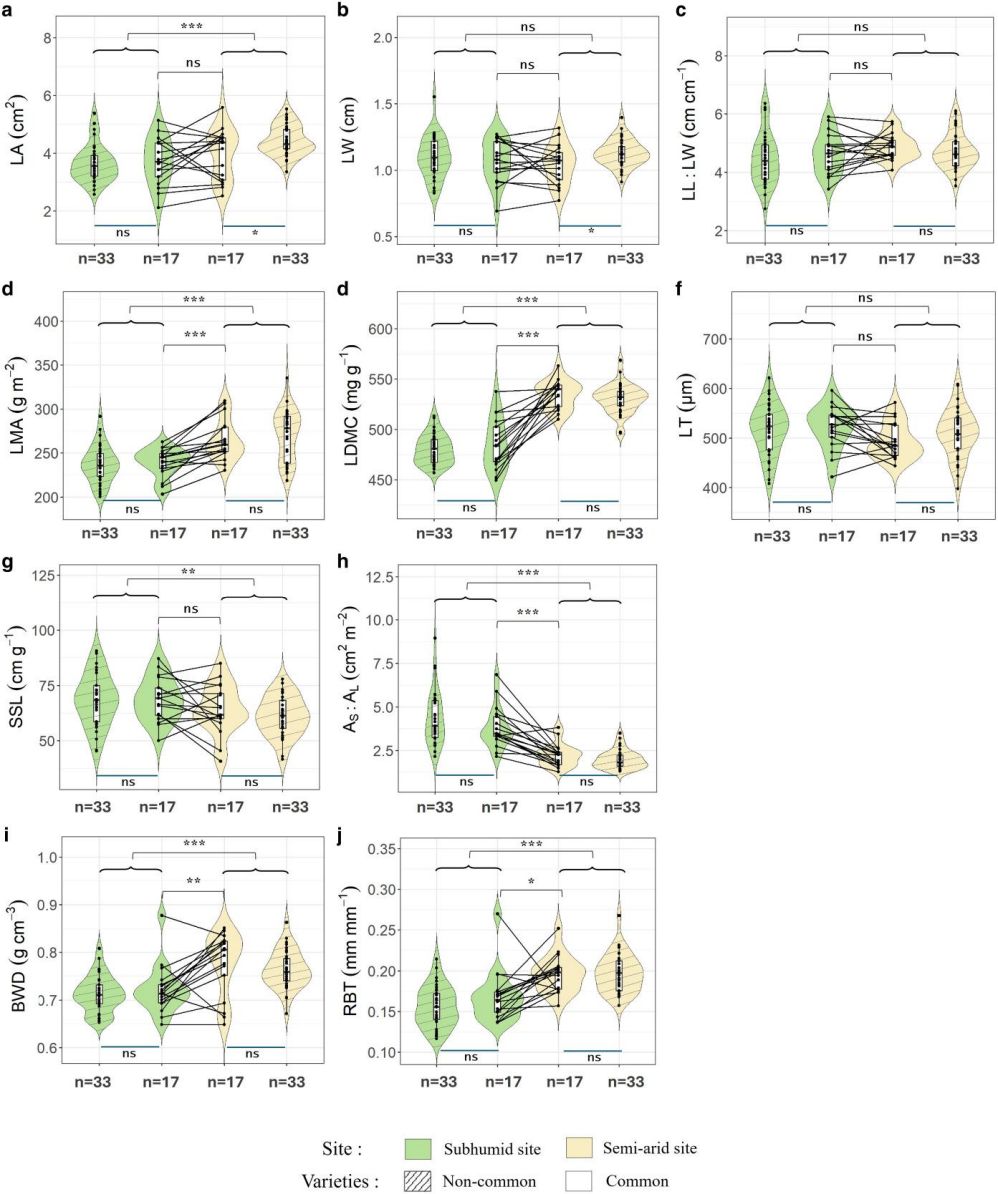
Variations in leaf (a–f), stem (g—and h), and branch (i and j) traits among the 83 olive varieties in the two sites. The black dots represent the mean trait values of varieties and the black lines indicate the trait variations for the common varieties between the two sites. The horizontal width of the violin reflects the density of the data. Differences between sites for common varieties and the entire set of varieties, as well as the differences between distinct and common varieties in each site are shown (***P < .001; **P < .01; *P < .05; ns = non-significant). Trait abbreviations: LA, leaf area; LW, leaf width; LL:LW, leaf length to width ratio; LMA, leaf mass per area; LDMC, leaf dry matter content; LT, leaf thickness; SSL, specific stem length; *A*_s_:*A*_l_, sapwood area to twig leaf area; BWD, branch wood density; RBT, relative bark thickness.

Trait variability was slightly higher across the 50 varieties studied in the subhumid site as compared to those studied in the semi-arid site for most traits, except for LMA and BWD (Fig. 2; see Supplementary Table S4): the average coefficients of variation calculated for the 10 measured traits were 14.8% for the set of varieties in the subhumid site and 12.4% for the varieties in the semi-arid site. At both sites, the two most variable traits were *A*_S_:*A*_L_ and LA, and the two least variable ones were LDMC and BWD. Overall, the average trait values and trait ranges were comparable for the distinct and common varieties (Fig. 2). The only exceptions were for the size-related traits LA and LW in semi-arid site (Fig. 2a and b), where the distinct varieties were found to have larger leaves than the common varieties.

Notwithstanding this large variation within varieties (see Supplementary Table S6), the site effect was well-pronounced for most traits, except for LW, LL:LW, and LT (Fig. 2). A striking result is that the site effect on trait values was generally consistent for the common varieties (*n* = 17) and for the entire set of varieties (*n* = 50). This holds for all traits, except for LA and SSL (Fig. 2): distinct varieties in the semi-arid site had larger leaves (Fig. 2a) and denser stems (Fig. 2g) than distinct varieties in the subhumid site.

The differences between sites were significant for traits related to leaf and wood structure, with trees in semi-arid site exhibiting higher LMA, LDMC, BWD and RBT (Figs. 2d and e, i, j). Conversely, *A*_S_:*A*_L_ was higher in the subhumid site (4.17 cm^2^ m^−2^) compared to the semi-arid site (2.06 cm^2^ m^−2^) (Fig. 2h). These traits showed considerable plasticity contributions (8.63%–39.17%).

Consistent phenotypic plasticity was observed for the common varieties, with nearly all varieties exhibiting a similar trend (direction) for plastic variation in LMA, LDMC and *A*_S_:*A*_L_ (Fig. 2d, e and h). For BWD and RBT, the trend was reversed for three varieties, only one of which is identical for both traits (‘Bouteillan’) (Fig. 2i and j). However, the magnitude of plasticity varied among varieties, reflecting significant ‘site × variety’ interactions (see Supplementary Table S5).

### Plasticity indices PhD and MVPi

**Figure 3.**
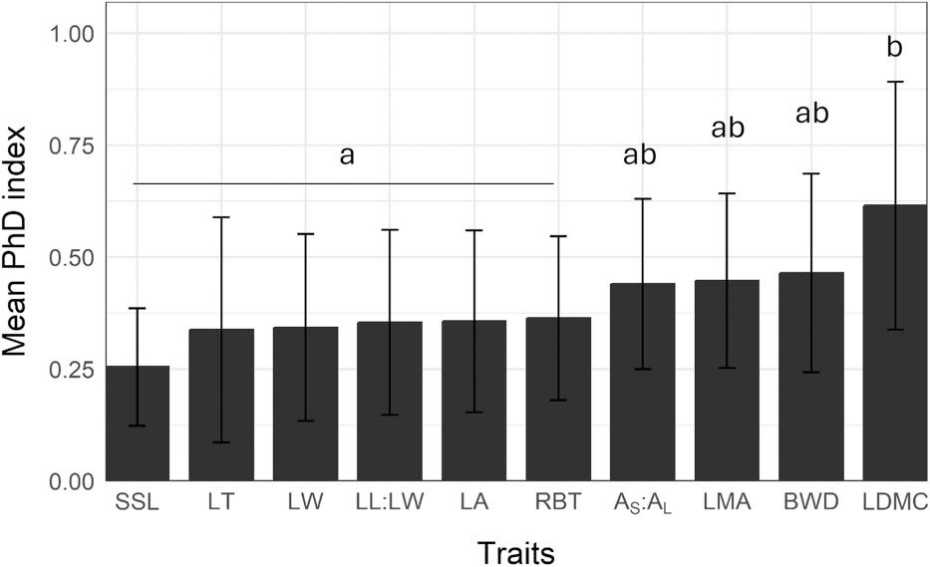
Bar graph showing the mean PhD for the different traits. Different letters indicate significant differences in mean PhD indices among traits (*P*-value < .05) using post-hoc tests. Trait abbreviations: LA, leaf area; LW, leaf width; LL:LW, leaf length to width ratio; LMA, leaf mass per area; LDMC, leaf dry matter content; LT, leaf thickness; SSL, specific stem length; *A*_s_:*A*_l_, sapwood area to twig leaf area; BWD, branch wood density; RBT, relative bark thickness.

The average PhD values of each trait ranged from 0.25 to 0.61 (see Supplementary Table S7). LDMC showed the largest plasticity (0.61), alongside the structural traits BWD (0.46), and LMA (0.45), followed by *A*_S_:*A*_L_ (0.44), while SSL, LT and leaf size and form were the least plastic traits (Fig. 3).

**Figure 4.**
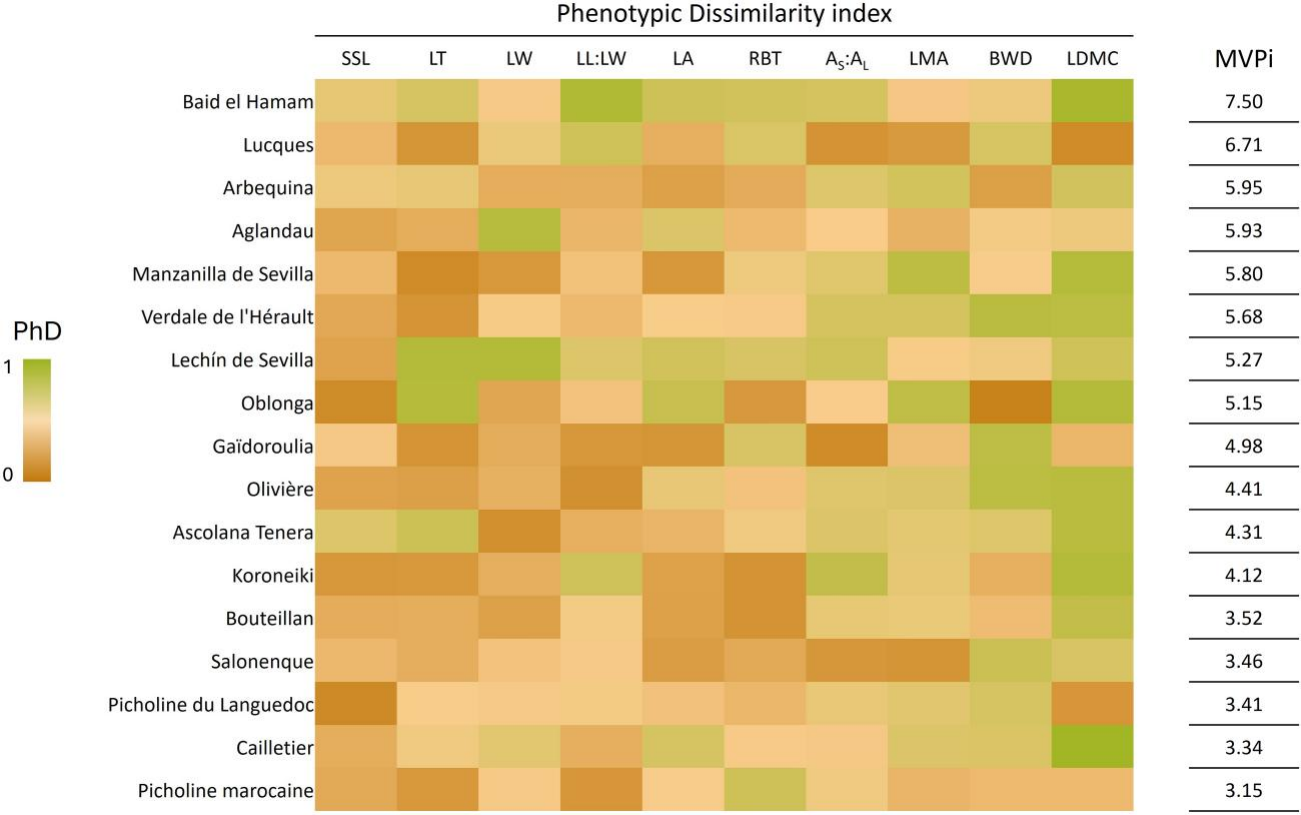
Heat map of the PhD. Varieties are ranked according to decreasing values of the MVPi, shown on the right-hand side of the heat map. Each cell represents the plasticity index of a specific variety for a given trait, with colour gradients indicating the range from 0 (no plasticity) to 1 (maximum plasticity) for the 17 common varieties. Trait abbreviations: LA, leaf area; LW, leaf width; LL:LW, leaf length to width ratio; LMA, leaf mass per area; LDMC, leaf dry matter content; LT, leaf thickness; SSL, specific stem length; *A*_s_:*A*_l_, sapwood area to twig leaf area; BWD, branch wood density; RBT, relative bark thickness.

The MVPi varied between 3.15 (‘Picholine marocaine’) and 7.50 (‘Baid El Hamam’) (Fig. 4). A key feature is that varieties with similar MVPi may show different levels of individual trait plasticity (Fig. 4; Supplementary Table S7). For instance, the most plastic variety ‘Baid El Hamam’, exhibited high plasticity in terms of LDMC and LL:LW, with moderate plasticity for the other traits. In contrast, ‘Lucques’, the second most plastic variety, showed low plasticity for LDMC, while its plasticity for BWD was relatively high (Fig. 4).

### Plasticity and resource use

**Figure 5.**
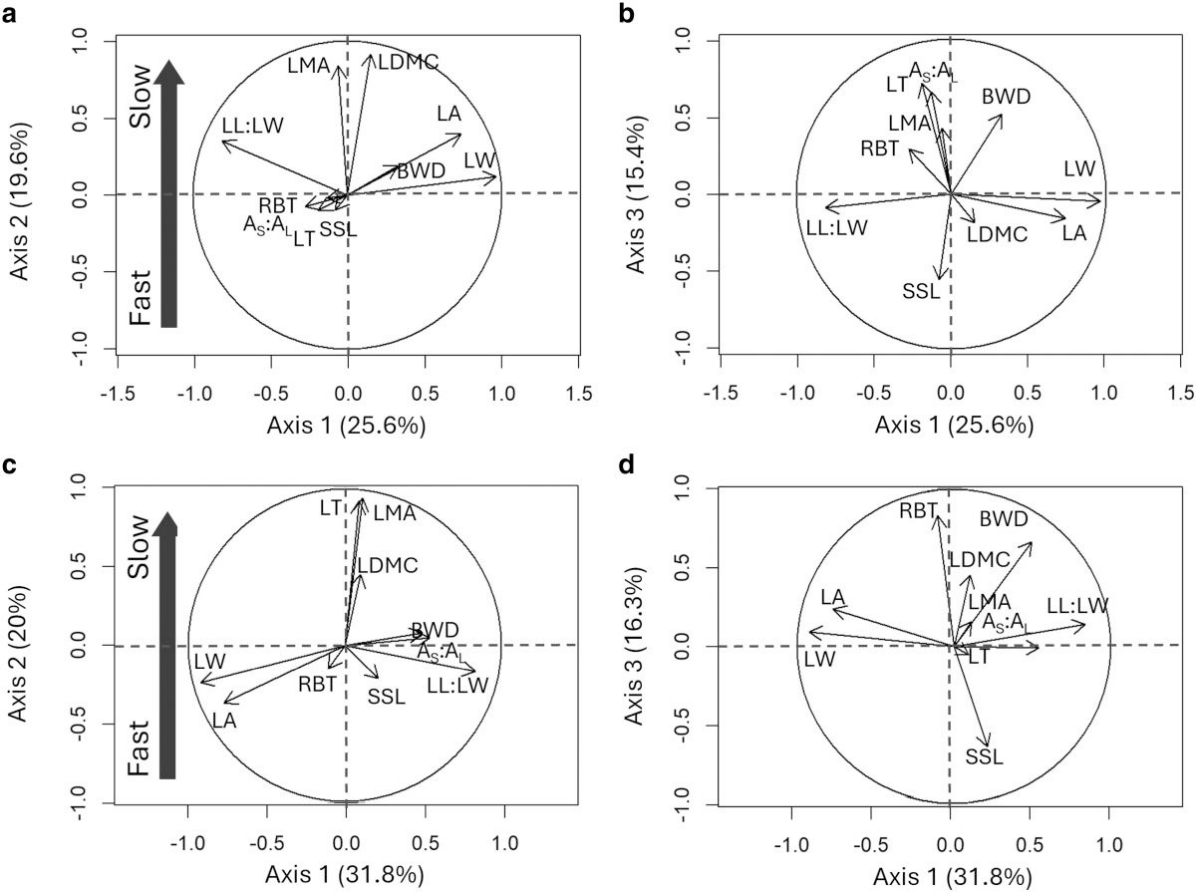
The first (axes 1 and 2) and second (axes 1 and 3) planes of principal component analyses (PCA) conducted on the 17 common varieties. a and b are for the subhumid site; c and d are for the semi-arid site. The black arrows indicate the direction and weighting of the vectors representing the traits under consideration. The large black arrow on the left-hand side of the correlation circles in a and c symbolizes the fast-slow axis of the PCAs. Trait abbreviations: LA, leaf area; LW, leaf width; LL:LW, leaf length to width ratio; LMA, leaf mass per area; LDMC, leaf dry matter content; LT, leaf thickness; SSL, specific stem length; *A*_s_:*A*_l_, sapwood area to twig leaf area; BWD, branch wood density; RBT, relative bark thickness.

The principal component analyses (PCAs) conducted on each of the two sites revealed slight differences in the correlation patterns among traits (Fig. 5; Supplementary Table S8). The first three axes accounted for 60.6% of the total variance in the subhumid site (Fig. 5a and b) and 68.1% in the semi-arid site (Fig. 5c and d). In both cases, the first axes (25.6% in the subhumid site, 31.8% in the semi-arid site) primarily discriminated varieties based on leaf form and size. This axis can be interpreted as a key dimension of leaf energy balance, given the crucial role of leaf size and form in the regulation of water loss and temperature (Wright et al. 2017). The second axis (19.6% in the subhumid site, 20% in the semi-arid site) reflected differences in leaf structure, particularly LMA and LDMC, and captured the ‘fast-slow’ axis of variation in both sites. The third axes (15.4% in subhumid site, 16.3% in semi-arid site), corresponded to differences in twig and branch traits, with a substantial contribution of BWD and SSL (see Supplementary Table S8). RBT was significantly associated with the third axis in the semi-arid site, but this association was weaker in the subhumid site (Fig. 5b and d).

**Figure 6.**
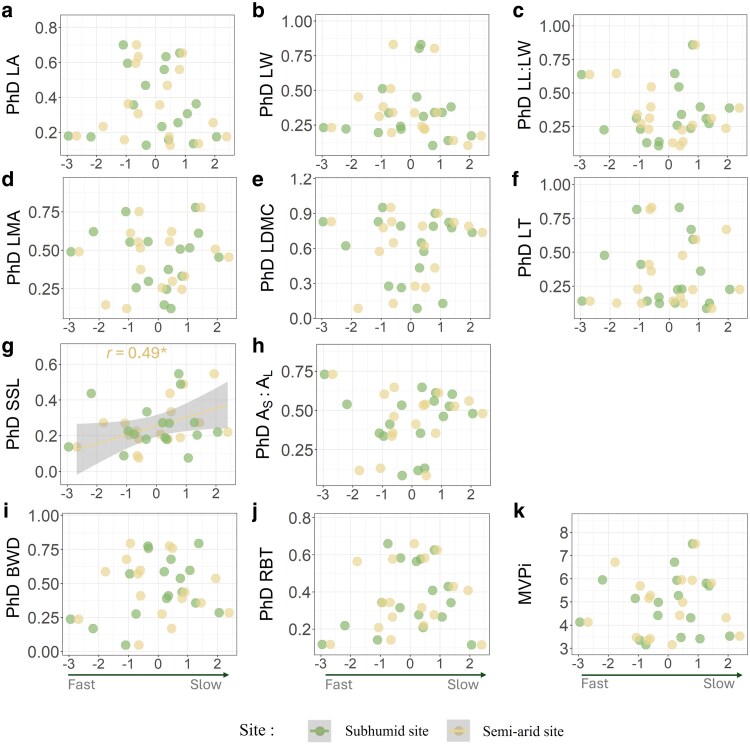
Relationships between the scores of varieties on the fast-slow axis (axis 2) of the PCAs presented in Fig. 5A–C and their plasticity assessed by the PhD index for each trait (a–j) and the MVPi index (k), assessed separately for the two collections. Varieties are represented by distinct dots for the subhumid and semi-arid sites. The only statistically significant relationship (*P < .05 for semi-arid site in g) is represented by a solid line, while the shadowed area shows confidence intervals (95%). Trait abbreviations: LA, leaf area, LW = leaf width, LL:LW = leaf length to width ratio, LMA = leaf mass per area, LDMC = leaf dry matter content, LT = leaf thickness, SSL = Specific stem length, *A*_s_:*A*_l_ = Sapwood area to twig leaf area, BWD = Branch wood density; RBT, relative bark thickness.

To assess whether a trade-off exists between plasticity and resource-conservation strategies, we investigated the relationship between the scores of varieties on the ‘fast-slow’ axes of the PCAs (see Supplementary Figure S1), and the Phenotypic Dissimilarity index for different traits on the one hand, and the MVPi on the other hand. The only significant correlation identified was a positive association between the ‘fast-slow’ score and the plasticity of SSL in the semi-arid site (Fig. 6g). No significant correlation was found with the MVPi in either the subhumid site or semi-arid site.

## DISCUSSION

The study of ten leaf and stem traits of olive varieties across two collections with contrasting environmental conditions, one in France (subhumid site) and the other one in Morocco (semi-arid site), showed substantial variations for all traits within each site. In the semi-arid site, higher temperatures also imply a much higher vapour pressure deficit and consequently stronger atmospheric drought stress independently of irrigation, which revealed consistent environmental effects on both the inter- and intra-varietal levels for most traits, but not systematically towards more conservative values compared to the subhumid site. Our results also reveal substantial differences in plasticity among traits and varieties, underscoring the complexity of assessing plasticity when several traits are considered simultaneously.

### Trait variations across olive varieties

Leaf size variation (area, length, width) across varieties compares well with what was observed in two other olive varietal collections (Cantini et al. 1999, Trentacoste and Puertas 2011), although some varieties certainly have larger leaves than those studied here: e.g. ‘Kalamon’ -not studied here-, has leaves 1.3 times larger than ‘Zard’, the variety with the largest leaves in our study: Koubouris et al. 2018. LMA values (218.6–335.4 g m^−2^) compare well with those found in other studies (e.g. Bacelar et al. 2004, Trentacoste et al. 2018). To the best of our knowledge, our study is the first to provide values for the other traits for olive varieties.

LMA values of olive were higher than those of other woody crops, both deciduous [e.g. wine grapes (*Vitis vinifera* L.: 52.8–101.8; Martin et al. 2025)] and evergreen [e.g. coffee (*Coffea arabica*: 52.9–142.9 g m^−2^; Martin and Isaac 2021, *Citrus* species.: 110.9–147.0; Romero-Trigueros et al. 2017)]. Based on the high average LMA value of olive relative to both cultivated and wild species (Wright et al. 2004), the olive tree can be considered as a ‘conservative’ species in the context of the leaf economics spectrum (see also discussion in Garnier et al. 2025). Similarly, values of wood density (0.64–0.86 g cm^−3^) are in the high range compared to other species (Chave et al. 2009, Díaz et al. 2016), suggesting that olive has a relatively high resistance to embolism, especially among woody crops. The substantial range of within-species variation in these traits found here nonetheless leads us to the conclusion that different olive varieties might be fit to different environments.

### Trait variation under contrasting environmental conditions

Although there was considerable variation in trait values among varieties within each site, the differences observed between sites for the 17 common varieties were generally similar to those seen across all varieties at each site. This indicates that, despite differences in varietal composition, environmental conditions exert a strong and consistent influence on trait values. A comparable pattern was also reported by Sauvadet et al. (2021) in cacao clonal gardens established under contrasting climates. In their study, morphological trait differences among clones were relatively consistent across sites, while chemical traits showed stronger site-driven variation. This contrast highlights that, in our case, environmental conditions had a marked effect even on morphological and anatomical traits, whereas in cacao, site effects were more evident for chemical traits.

Although smaller and thinner leaves are generally expected under more arid conditions (Wright et al. 2017), this was not observed here: leaves had similar dimensions (leaf width and elongation) in both sites (Fig. 2). The mean leaf area was even found to be higher in the semi-arid site where aridity and temperature were higher, but this difference was mainly explained by the leaf size of some varieties with particularly big leaves (e.g. ‘Carolea’) that were not present in the subhumid site and therefore cannot be directly attributed to the environment. Overall, the results suggest a high phenotypic stability of leaf size and dimensions among olive varieties in response to environmental conditions. This contrasts with the results reported in wild olive (*O. europaea* subsp. *europaea* var. *sylvestris*) along a natural environmental gradient in Morocco, where leaves were found to become smaller and more elongated as aridity increased (Kassout et al. 2024).

In contrast, olive trees exhibited denser leaves with higher LMA, higher BWD, and thicker bark in the semi-arid site compared to the subhumid site. The contrasting climatic conditions between these two sites, notably differences in temperature and aridity, are likely key factors driving behind these phenotypic variations. Higher LMA values resulted solely from increased LDMC, as there was no difference in leaf thickness between the two sites, contrary to expectations from previous studies (e.g. Niinemets 2001). Leaves with high LDMC, and/or tissue density, have been found to have a low osmotic potential at the turgor loss point (Niinemets 2001, Májeková et al. 2021, Nadal et al. 2023), which is considered a key factor in drought tolerance. Similarly, a high BWD has been associated with a higher resistance to cavitation (Chave et al. 2009, Reich 2014 and references therein). This aligns with a recent study on olive (Terral et al. 2025), which shows that varieties originating from arid areas have smaller vessels at a given branch diameter and, therefore, likely a higher BWD (Fajardo et al. 2022) than varieties from more humid environments. Interestingly, the higher wood density in the semi-arid site was not associated to lower SSL. This may be explained by the small diameter, resulting from slower growth of twigs in this collection (mean ± SD: 1.01 ± 0.15 mm in the semi-arid site vs. 1.19 ± 0.21 mm in the subhumid site; ANOVA: *F* = 24.39, *P* < 0.001) where the environmental conditions are harsh. Thicker bark is also expected in drier sites with high temperatures, as it confers enhanced water storage and protection (Rosell et al. 2014). To the best of our knowledge, our study is among the first to show such a response of bark thickness at the intraspecific level.

The sapwood area to twig leaf area ratio (*A*_S_:*A*_L_) was lower at the semi-arid site, which was unexpected and contrasts with studies conducted on gymnosperms (DeLucia et al. 2000, Mencuccini and Bonosi 2001). However, using a large data set of 1135 tree species, Mencuccini et al. (2019) found only weak relationships between the *A*_S_:*A*_L_ ratio and mean annual precipitation or site aridity, and no significant relationship with mean annual temperature. This ratio appears to be influenced by a complex combination of other traits, including LMA and specific hydraulic conductivity (*K*_s_) (Mencuccini et al. 2019), which may vary in different directions at the intraspecific level and compensate for each other. In fact, trees with a lower *A*_S_:*A*_L_ ratio tend to have a higher *K*_s_, allowing them to maintain an adequate water supply to the leaves despite their lower relative sapwood area.

### Plasticity across olive varieties

The relative contributions of phenotypic plasticity compared to genetic variation varied across traits, ranging from 0.17% to 39.17%. Traits differed significantly in their overall degree of plasticity. Structural and density traits (LMA, LDMC, BWD) were the most plastic, and showed a marked response to the environment, although a few varieties displayed opposite patterns. In contrast, leaf and stem size traits were relatively less plastic, indicating a clear distinction between different types of traits in response to the environment. Interestingly, within the common varieties the two most plastic traits (LDMC and LMA) between sites were those displaying the smallest variation among varieties, suggesting some independence between the processes underlying trait variation. However, this general conclusion hides striking differences among varieties: e.g. a variety that is overall described as ‘not very plastic’ (i.e. ‘Picholine marocaine’; MVPi = 3.15) may actually display high plasticity for certain traits (e.g. RBT; PhD index = 0.66). In contrast, a variety that showed higher overall plasticity (i.e. ‘Lucques’; MVPi = 6.71) had much lower plasticity across most traits (e.g. LDMC; PhD index = 0.09). This raises two unsolved issues for the study of plasticity. On the one hand, using a single composite metric to assess plasticity (such as MPVi) may obscure the underlying mechanisms of acclimation, which might be better captured by using univariate metrics focused on a targeted, relevant trait. On the other hand, as a plant's response to environmental variation actually involves multiple traits (Pennacchi et al. 2021, Anderegg 2023), a multivariate index might be more appropriate to assess the fit between an organism and its environment. Addressing these issues requires a better understanding of how different trait combinations and plastic responses impact fitness, or fruit yield in the case of olive, in specific environments. Unfortunately we lack such data in our study, making it difficult to clearly evaluate how the observed plasticity leads to improved performance. Future work should integrate yield-related traits in order to better evaluate whether plastic responses confer a tangible agronomic benefit. This will be essential to identify the most valuable plastic varieties, i.e. those capable of maintaining high and stable performance under environmental stress (Brooker et al. 2022).

Finally, more ‘conservative’ varieties are often assumed to be less plastic than those that are more ‘acquisitive’ (Grime 1977, Grassein et al. 2010, Stotz et al. 2022, Carrascosa et al. 2023). This is likely because more conservative varieties (like any plant) tend to prioritize survival over growth, in relation to high tissue construction costs and slower tissue renewal (Wright et al. 2004, Poorter et al. 2009), leading to limited plasticity. One possible explanation is that phenotypic plasticity itself comes with metabolic and resource costs. As proposed by DeWitt et al. (1998), rapid phenotypic reconfiguration requires substantial energetic investment, which may be incompatible with a resource-conserving strategy. Several studies have reported this trade-off between stress tolerance and phenotypic plasticity (Power et al. 2019, Solé-Medina et al. 2022). In their interspecific analysis based on a literature survey involving 61 woody evergreens, Stotz et al. (2022) found that SLA (the inverse of LMA), a major trait contributing to the fast-slow axis, was positively related to plasticity in plant biomass allocation and physiology (mainly net photosynthetic rate), but negatively related to plasticity in plant size, and unrelated to plasticity in leaf morphology. As discussed above, this highlights the multidimensional nature of phenotypic plasticity and the paramount importance of trait selection when assessing plasticity. Our results for olive—an evergreen sclerophyll species—are in line with those of Stotz et al. (2022) for leaves and extend their conclusions to stem and branch traits. The only evidence we found of a relationship between the fast-slow axis plasticity was for SSL, and it was contrary to our expectations: SSL was more plastic in more conservative varieties. We might argue that the range of variation in LMA values (which varies approximately twofold across the studied varieties) was too small to detect any relationship in our study. However, the consistency between our results and the literature survey conducted by Stotz et al. (2022) across species with a larger range of variation in LMA instead suggests that phenotypic plasticity relates only poorly to plant resource use strategy (see also Garnier et al. 2019).

### Towards an olive breeding programme integrating phenotypic plasticity

Using the natural phenotypic diversity of olive is a strategic lever for varietal selection programmes, particularly in the context of climate change. This diversity, which includes phenotypic plasticity, enables the assessment of the ability of varieties to cope with contrasting environments. By studying phenotypic traits linked to the water and carbon use in two *ex situ* varietal collections with very contrasting environmental conditions, we were able to identify traits with a high degree of plasticity, providing a basis for exploring how these plastic responses could influence the olive varieties to persist under harsh conditions. At the same time, stable traits across environments reflect genetic robustness. This is the case, e.g. leaf thickness, which is functionally relevant but invariant across sites. These ‘invariant’ traits are deemed to have little relevance for breeding under climate change. Our study suggests that the focus should instead be directed towards structural and density related traits of leaves and/or stems.

## Conclusions

Although genetic variation among varieties was substantial, phenotypic plasticity contributed considerably to trait variability (Q1). Our results reveal distinct phenotypic signatures at the two sites, with trait variations globally supporting reduced water loss at the drier site. The direction of changes in trait values was generally consistent among and within varieties for most leaf and stem traits, and especially for density traits (LMA, LDMC, BWD), suggesting that plastic changes in these traits could be considered as the initial steps of an acclimation response in olive. Comparing plasticity among varieties remains challenging due to the complexity of assessing plasticity beyond individual traits (Q2–Q3). A better understanding of how traits combine to impact production in olive would help assess the role of multivariate plasticity in shaping plant responses to aridity. However, we did not find any evidence that more conservative olive varieties were less plastic than more acquisitive ones (Q4). In the context of unpredictable climate change, breeding varieties based on plastic traits associated with water use could be a key lever for ensuring the sustainability of crops in the Mediterranean Basin.

## Supplementary Material

plaf060_Supplementary_Data

## Data Availability

The values of leaf, stem, and branch traits averaged for each tree for all varieties are archived at the CNRS institutional repository InDoRES (https://data.indores.fr:443/dataverse/CEFE) with the following DOI: https://doi.org/10.48579/PRO/OQZOB9 (Wakib et al., 2025).
