# Supplementary material for: Intraspecific variation and phenotypic plasticity of olive varieties in response to contrasting environmental conditions
Source: AoB Plants. 2025 Nov 13;17(6):plaf060. doi: 10.1093/aobpla/plaf060 (PMC12612625; doi:10.1093/aobpla/plaf060)
Supplement: plaf060_Supplementary_Data [file plaf060_supplementary_data.pdf]

Table S1. Soil characteristics of the two studied olive collections (EC = Electrical conductivity, C/N = Carbon to nitrogen ratio).

	Soil pH	Soil texture	EC	Organic matter	Nitrogen	C/N
			($mS\ cm^{-1}$)	(%)	(%)	
Subhumid site	6.55	Sandy loam	0.07	1.21	0.06	11.50
Semi-arid site	7.42	Silt loam	0.34	0.88	1.07	8.13

Table S2. Name, origin, genetic pool and usage of the distinct varieties (n=33) studied in each of the two olive collections (subhumid and semi-arid sites). Usage data were obtained from the OLEA databases (https://oleadb.it/).

Subhumid site				Semi-arid site			
Name	Origin	GenePool	Usage	Name	Origin	GenePool	Usage
Amellau	France	Eastern	Oil, table	Aaleth	Algeria	Central	Oil
Amigdalolia	Greece	Central	Oil, table	Adkam	Syria	Eastern	Unknown
Aubenc	France	Unknown	Oil	Aggezi Oshime	Egypt	Central	Table
Baguet	France	Admixed	Oil	Azeradj	Algeria	Eastern	Oil, table
Barouni	Tunisia	Western	Oil, table	Barouni	Tunisia	Western	Oil, table
Béchude	France	Admixed	Oil	Bed Al Iguel	Syria	Eastern	Unknown
Blanche de Paysac	France	Eastern	Oil	Beladi	Liban	Eastern	Oil, table
Cayet roux	France	Admixed	Oil, table	Carolea	Italy	Eastern	Oil, table
Cayon	France	Admixed	Oil, table	Cayon	France	Admixed	Oil, table
Chemlali	Tunisia	Admixed	Oil, table	Chemlal de Kabilye	Algeria	Central	Oil
Colombale	France	Admixed	Oil	Coratina	Italy	Central	Oil, table
Courbeil	France	Admixed	Oil	Dhokar	Tunisia	Central	Oil
Cul Blanc	France	Admixed	Unknown	Farga	Spain	Central	Oil
Curnet	France	Eastern	Oil	Fouji vert	Tunisia	Eastern	Oil, table
Filayre rouge	France	Eastern	Unknown	Galega Vulgar	Portugal	Central	Oil, table
Gardisson	France	Admixed	Unknown	Grappolo	Italy	Central	Oil
Grapié	France	Admixed	Oil	Hojiblanca	Spain	Western	Oil, table
Grappola	Italy	Central	Oil, table	Ibleb	Syria	Eastern	Unknown
Grassois	France	Central	Oil	Itrana	Italy	Admixed	Oil, table
Gros vert	France	Central	Unknown	Kato Drys	Cyprus	Eastern	Oil, table
Grossane	France	Admixed	Oil, table	Leccino	Italy	Central	Oil, table
Moufla	France	Eastern	Oil	Manzanilla Cacereña	Spain	Western	Oil, table
Moutaurounenque	France	Admixed	Oil	Manzanilla de Agua	Spain	Western	Table
Négrette des Vans	France	Admixed	Oil	Meslala	Morocco	Western	Oil, table
Petit Ribier = Rabeyrolle	France	Central	Oil	Moraiolo	Italy	Central	Unknown
Picual	Spain	Western	Oil, table	Picual	Spain	Western	Oil, table
Reymet	France	Centre	Oil	Sevillenca	Spain	Admixed	Oil
Rougette de l'Ardèche	France	Admixed	Oil	Tabelout	Algeria	Central	Oil
Rougette du Gard	France	Admixed	Unknown	Tarabelsi	Syria	Admixed	Unknown
Sauzen Vert	France	Admixed	Unknown	Toffahi	Syria	Eastern	Table
Tanche	France	Eastern	Oil, table	Wateken	Egypt	Admixed	Oil, table
Verdanel	France	Admixed	Oil	Zaity	Syria	Eastern	Oil, table
Zard	Iran	Eastern	Oil, table	Zarza	Spain	Admixed	Oil

Table S3. Name, origin, genetic pool and usage of the common varieties (n=17) studied in the two olive collections. Usage data were obtained from the OLEA databases (https://oleadb.it/).

Name	Origin	GenePool	Usage
Aglandau	France	Eastern	Oil, table
Arbequina	Spain	Central	Oil
Ascolana Tenera	Italy	Admixed	Oil, table
Baid El Hamam (= Meski)	Egypt	Admixed	Oil, table
Bouteillan	France	Admixed	Oil, table
Cailletier	France	Admixed	Oil, table
Lechín de Sevilla (= Ecijano)	Spain	Admixed	Oil
Gaïdourolia (= Chalkidikis)	Greece	Admixed	Oil, table
Koroneiki	Greece	Central	Oil
Lucques	France	Admixed	Oil, table
Manzanilla de Sevilla	Spain	Western	Oil, table
Picholine marocaine (= Menara)	Morocco	Western	Oil, table
Oblonga (= Frantoio)	Italy	Central	Oil
Olivière	France	Admixed	Oil, table
Picholine du Languedoc	France	Admixed	Oil, table
Salonenque	France	Eastern	Oil, table
Verdale de l'Hérault	France	Admixed	Oil, table

Table 4. Descriptive statistics for leaf and stem traits of the 50 varieties studied in each site.

	Subhumid site				Semi-arid site			
	Mean (SE)	Range	CV (%)	Number of replicates	Mean (SE)	Range	CV (%)	Number of replicates
LA (cm^2)	3.66 (0.10)	5.38-2.11	19.30	500	4.26 (0.09)	5.58-2.52	16.29	600
LW (cm)	1.08 (0.02)	1.55-0.69	14.61	500	1.10 (0.01)	1.39-0.77	11.51	600
LL:LW (cm cm^{-1})	4.52 (0.11)	6.35-2.74	18.53	500	4.77 (0.07)	6.10-3.52	11.80	600
LMA (g m^{-2})	237.86 (2.76)	291.75-222.13	8.22	500	268.19 (3.81)	335.38-218.59	10.04	600
LDMC (mg g^{-1})	481.62 (2.66)	513.30-463.47	3.90	500	531.65 (2.06)	543.52-512	2.74	600
LT (µm)	518.58 (6.87)	520.69-407.56	9.36	500	504.15 (6.46)	607.97-397.25	9.07	600
SSL (cm g^{-1})	68.31 (11.04)	90.59-45.26	16.17	300	62.06 (9.9)	84.86-40.63	15.95	300
A_S:A_L (cm^2 m^{-2})	4.17 (1.44)	8.95-2.14	34.5	300	2.06 (0.59)	3.82-1.26	28.7	300
BWD (g cm^{-3})	0.71 (0.003)	0.87-0.64	6.02	300	0.76 (0.004)	0.86-0.64	6.57	300
RBT (mm mm^{-1})	0.16 (0.02)	0.26-0.11	17.5	300	0.19 (0.02)	0.26-0.15	11.7	300

CV, coefficient of variation.
Trait abbreviations: LA = Leaf area, LW = Leaf width, LL:LW = Leaf length to width ratio, LMA = Leaf mass per area, LDMC = Leaf dry matter content, LT = Leaf thickness, SSL = Specific stem length, A_s:A_l = Sapwood area to twig leaf area, BWD = Branch wood density, RBT = Relative bark thickness.

Table S5. Two-Way ANOVA results for the 17 common varieties (n=17). F-values are shown for the effects of Site, Variety and their interaction (Site x Variety) on the measured traits. Analyses were conducted on replicate trees.. Significance levels are indicated : ***p < 0.001; **p < 0.01; *p < 0.05.

	n=17		
	Site	Variety	Site x Variety
LA (cm^2)	8.81**	14.6***	5.45***
LW (cm)	1.5	16.02***	6.74***
LL:LW ($cm\ cm^{-1}$)	8.98**	6.96***	4.68***
LMA ($g\ m^{-2}$)	116.8***	11.62***	3.45***
LDMC ($mg\ g^{-1}$)	289.64***	6.76***	5.26***
LT (µm)	28.46***	10.64***	4.85***
SSL ($cm\ g^{-1}$)	3.91	3.6***	1.94*
A_S:A_L ($cm^2\ m^{-2}$)	75.8***	4.16***	2.38**
BWD ($g\ cm^{-3}$)	55.55***	10.6***	7.02***
RBT ($mm\ mm^{-1}$)	32.18***	3.32***	4.29***

Trait abbreviations: LA = Leaf area, LW = Leaf width, LL:LW = Leaf length to width ratio, LMA = Leaf mass per area, LDMC = Leaf dry matter content, LT = Leaf thickness, SSL = Specific stem length, A_s:A_l = Sapwood area to twig leaf area, BWD = Branch wood density, RBT = Relative bark thickness.

Table S6. Relative contributions of genetic variation, phenotypic plasticity and residual variance to overall trait variation. Contributions were calculated from the sum of squares of the one-way ANOVAs. The table reports values for each trait, along with the mean (± SD), and the range.

	Percentage of contribution (%)		
	Genetic variation	Phenotypic plasticity	Residual variance
Leaf area (cm^2)	44.84	1.18	53.99
Leaf width (cm)	50.08	0.17	49.75
Leaf length to width ratio ($cm\ cm^{-1}$)	59.54	1.34	39.13
Leaf mass per area ($g\ m^{-2}$)	51.03	13	35.98
Leaf dry matter content ($mg\ g^{-1}$)	24.58	39.17	36.35
Leaf thickness (µm)	58.03	3.35	38.61
Specific stem length ($cm\ g^{-1}$)	44.75	1.35	53.9
Sapwood area to twig leaf area ($cm^2\ m^{-2}$)	44.48	16.93	38.6
Branch wood density ($g\ cm^{-3}$)	39.77	8.36	51.87
Relative bark thickness ($mm\ mm^{-1}$)	40.43	8.63	50.94
Mean (± SD)	45.75 ± 9.50	9.34 ± 11.3	44.91 ± 7.33
Range	24.58 – 59.54	0.17 – 39.17	36.35-53.99

Table S7. Phenotypic Dissimilarity index (PhD) measured for the 17 common varieties for the 10 traits.

	Phenotypic Dissimilarity index									
	SSL	LT	LW	LL:LW	LA	RBT	A_S:A_L	LMA	BWD	LDMC
Baid el Hamam	0.49	0.59	0.34	0.86	0.65	0.63	0.61	0.33	0.44	0.9
Lucques	0.27	0.13	0.45	0.64	0.23	0.56	0.12	0.15	0.59	0.09
Arbequina	0.44	0.48	0.22	0.23	0.17	0.22	0.54	0.62	0.17	0.62
Aglandau	0.19	0.23	0.8	0.26	0.56	0.28	0.36	0.25	0.39	0.43
Manzanilla de Sevilla	0.27	0.09	0.14	0.31	0.13	0.43	0.53	0.78	0.36	0.82
Verdale de l'Hérault	0.2	0.12	0.38	0.27	0.36	0.34	0.6	0.61	0.79	0.78
Lechín de Sevilla	0.18	0.83	0.83	0.55	0.63	0.58	0.65	0.38	0.41	0.65
Oblonga	0.09	0.82	0.20	0.31	0.7	0.14	0.36	0.75	0.05	0.83
Gaidoroulia	0.33	0.12	0.22	0.14	0.13	0.58	0.09	0.3	0.76	0.26
Olivière	0.18	0.17	0.24	0.11	0.47	0.31	0.53	0.56	0.77	0.79
Ascolana Tenera	0.55	0.67	0.1	0.24	0.26	0.41	0.56	0.51	0.54	0.79
Koroneiki	0.14	0.14	0.23	0.64	0.18	0.12	0.73	0.49	0.24	0.83
Bouteillan	0.22	0.23	0.17	0.39	0.17	0.11	0.48	0.46	0.29	0.74
Salonenque	0.27	0.23	0.31	0.34	0.16	0.21	0.13	0.12	0.68	0.57
Picholine du Languedoc	0.08	0.36	0.34	0.4	0.31	0.26	0.46	0.52	0.6	0.13
Cailletier	0.23	0.41	0.51	0.23	0.6	0.34	0.34	0.55	0.57	0.95
Picholine marocaine	0.21	0.14	0.34	0.13	0.36	0.66	0.41	0.26	0.28	0.28
Mean	0.25	0.34	0.34	0.35	0.36	0.36	0.44	0.45	0.46	0.61

Trait abbreviations: LA = Leaf area, LW = Leaf width, LL:LW = Leaf length to width ratio, LMA = Leaf mass per area, LDMC = Leaf dry matter content, LT = Leaf thickness, SSL = Specific stem length, A_s:A_l = Sapwood area to twig leaf area, BWD = Branch wood density, RBT = Relative bark thickness.

Table S8. Percentage of variance explained, eigenvalues and loadings of principal components (PC1, PC2 and PC3) for both PCAs.

	PCA for subhumid site			PCA for semi-arid site		
	PC1	PC2	PC3	PC1	PC2	PC3
**Variation explained**	25.6	19.6	15.4	31.8	20	16.3
**Eigenvalue**	2.56	1.96	1.54	3.18	2	1.63
Variable loadings						
Leaf area (cm^2)	**0.73**	0.39	-0.16	**-0.76**	-0.37	0.23
Leaf width (cm)	**0.96**	0.11	-0.04	**-0.92**	-0.23	-0.09
Leaf length to width ratio ($cm\ cm^{-1}$)	**-0.81**	0.34	-0.08	**0.83**	-0.16	0.13
Leaf mass per area ($g\ m^{-2}$)	-0.05	**0.83**	0.42	0.11	**0.93**	0.15
Leaf dry matter content ($mg\ g^{-1}$)	0.15	**0.90**	-0.18	0.09	0.45	0.45
Leaf thickness (µm)	-0.18	-0.09	**0.70**	0.09	**0.91**	-0.05
Specific stem length ($cm\ g^{-1}$)	-0.07	-0.09	**-0.55**	0.21	-0.21	**-0.64**
Sapwood area to twig leaf area ($cm^2\ m^{-2}$)	-0.13	-0.02	**0.65**	**0.53**	0.04	-0.01
Branch wood density ($g\ cm^{-3}$)	0.32	0.18	**0.51**	0.49	0.07	**0.66**
Relative bark thickness ($mm\ mm^{-1}$)	-0.27	-0.07	0.28	-0.10	-0.15	**0.82**

Values greater than 0.5 are in bold

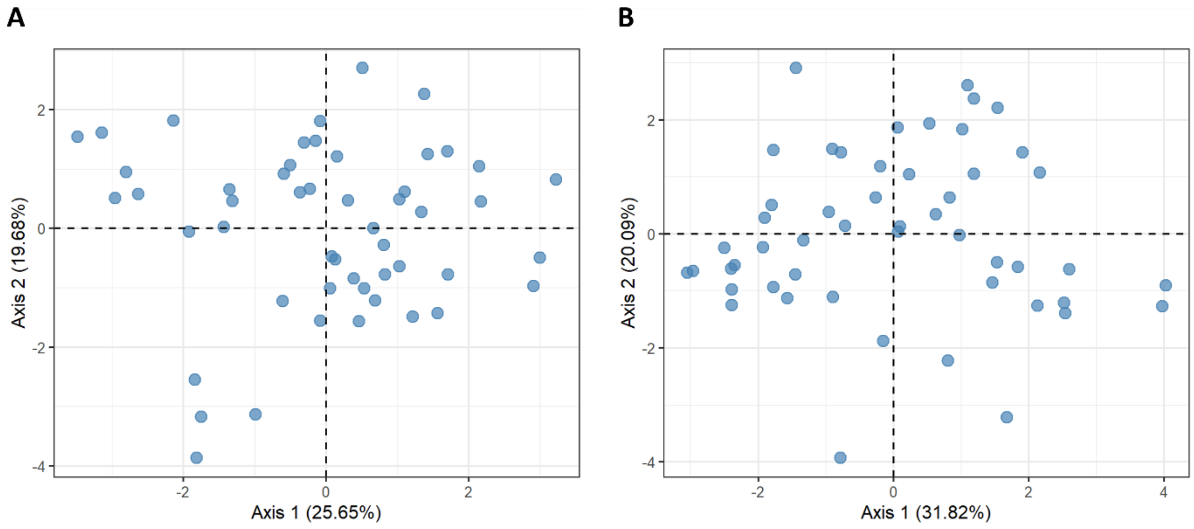

Figure S1. Principal component analysis (PCA) of the 17 common varieties, showing the first two principal component axes. Panels A and B correspond to the subhumid and semi-arid sites, respectively. Blue points represent individual trees.
